# Dynamic regulation of PGC-1α localization and turnover implicates mitochondrial adaptation in calorie restriction and the stress response

**DOI:** 10.1111/j.1474-9726.2007.00357.x

**Published:** 2008-02

**Authors:** Rozalyn M Anderson, Jamie L Barger, Michael G Edwards, Kristina H Braun, Clare E O’Connor, Tomas A Prolla, Richard Weindruch

**Affiliations:** 1 University of Wisconsin Madison, GRECC VA Hospital 2500 Overlook Terrace, Madison, WI 53705, USA; 2 Wisconsin National Primate Research Center 1220 Capitol Court, Madison, WI 53706, USA; 3 Department of Genetics and Biotechnology 425 Henry Mall, Madison, WI 53706, USA

**Keywords:** GSK3β, longevity, mitochondria, oxidative stress, PGC-1α, SIRT1

## Abstract

There is increasing evidence that longevity and stress resistance are connected, but the mechanism is unclear. We report that mitochondria are regulated in response to oxidative stress and calorie restriction through a shared mechanism involving peroxisome proliferator-activated receptor-γ co-activator 1α (PGC-1α). We demonstrate that PGC-1α subcellular distribution is regulated, and its transcriptional activity is promoted through SIRT1-dependent nuclear accumulation. In addition, the duration of PGC-1α activity is regulated by glycogen synthase kinase beta (GSK3β), which targets PGC-1α for intranuclear proteasomal degradation. This mechanism of regulation permits the rapidity and persistence of PGC-1α activation to be independently controlled. We provide evidence that this pathway of PGC-1α regulation occurs *in vivo* in mice, both in the oxidative stress response and with calorie restriction. Our data show how mitochondrial function may be adapted in response to external stimuli, and support the concept that such adaptation is critically involved in cellular survival and in lifespan extension by calorie restriction.

## Introduction

Cellular response to stress generally reflects a balance between cell survival and death. Stress resistance is a measure of the cell's ability to survive under conditions that are detrimental. Manipulations that extend lifespan often increase stress resistance at the cellular level, and a number of factors that play a role in the stress response have also been implicated in longevity. It is unclear if regulation of metabolism is a feature of cellular survival or how the metabolic state of the cell influences stress resistance. Mitochondria are the key organelle in substrate utilization and energy production. Transcriptional profiling studies demonstrate that genes involved in mitochondrial energy metabolism are coordinately up-regulated in multiple tissues with calorie restriction (CR), suggesting a change in dynamic of the electron transport system and a role for this alteration in mitochondrial metabolism in the mechanisms of CR (Lee *et al*., 1999, 2002). Biochemical analysis suggests that mitochondria from restricted tissues are functionally different from their control counterparts in terms of metabolism and composition (Bevilacqua *et al*., 2005; Hepple *et al*., 2005; Baker *et al*., 2006; Faulks *et al*., 2006). The factors involved in exerting these CR-dependent changes in mitochondrial function are unknown. Identification of these factors would provide insight into the mechanism of mitochondrial regulation in response to CR. Our understanding of the complexity of signalling pathways to and from the mitochondria is increasing (Horbinski & Chu, 2005; Jazwinski, 2005; Schieke & Finkel, 2006), describing a network through which mitochondria may communicate functional status to the nucleus to impact cellular function. Metabolic reprogramming by CR may be central to the mechanism of lifespan extension, where changes in mitochondrial function confer an energetic shift that is conducive to increased cellular fitness, resulting in the promotion of longevity (Anderson & Weindruch, 2007).

The transcriptional co-activator peroxisome proliferator-activated receptor-γ (PPAR-γ) co-activator 1α (PGC-1α) plays a multifaceted role in the regulation of metabolism (Puigserver & Spiegelman, 2003). PGC-1α regulates mitochondrial energy metabolism and biogenesis (Finck & Kelly, 2006), and influences carbohydrate and lipid utilization through co-activation of members of nuclear receptor family [e.g. peroxisome proliferator-activated receptor-α (PPAR-α), PPAR-γ, estrogen related receptor α (ERRα)] (Corton & Brown-Borg, 2005). PGC-1α is involved in skeletal muscle fibre type switching, responding to changes in oxidative demand in skeletal muscle (Lin *et al*., 2002), and is also induced with exercise (Baar *et al*., 2002), indicating that it is responsive to conditions of increased oxygen utilization. PGC-1α-deficient mice have defects in adaptive metabolism in that the metabolic response to hormonal stimuli, cold or the fasting state is dysfunctional in these animals (Lin *et al*., 2004; Leone *et al*., 2005). These data suggest that PGC-1α is a key component in coordinating the animal's metabolic response to external stimuli including nutritional status. Consistent with this, CR increases levels of PGC-1α mRNA in multiple tissues (Nisoli *et al*., 2005) and is induced in cells treated with serum from restricted animals (Lopez-Lluch *et al*., 2006), raising the possibility that PGC-1α is directly responsible for the mitochondrial changes induced by CR. We sought to identify factors involved in regulation of mitochondrial function, and determine if these factors could be connected with cellular survival or longevity pathways.

## Results

### Effect of PGC-1α on mitochondrial function

**Fig. 1:**
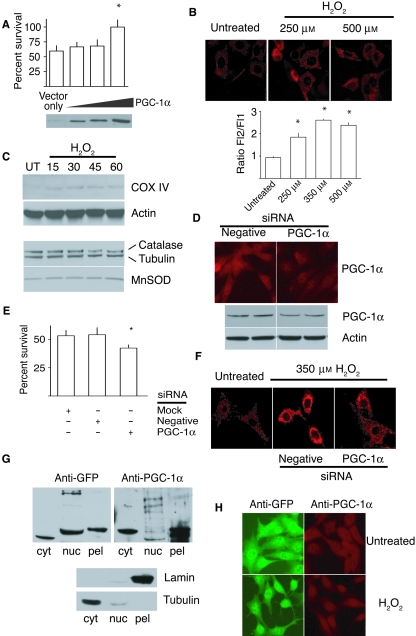
PGC-1α regulates mitochondrial function in response to stress. (A) Percent survival of NIH3T3 fibroblasts after treatment with H_2_O_2_ (350 µm); values represent means ± standard error of the mean (SEM); * indicates significant difference in survival compared to vector control (*P* < 0.05). (B) MitoTracker Red or JC-1 measurement of mitochondrial membrane potential in 1 h of H_2_O_2_treatment at indicated doses. Higher ratio of Fl2/Fl1 is indicative of higher membrane potential. Values represent means ± SEM; * indicates significant difference compared to untreated (*P* < 0.05). (C) Western analysis of COX IV, catalase and MnSOD in cell lysates taken at indicated times following exposure to hydrogen peroxide (350 µm). (D) PGC-1α RNA interference specifically reduces the level of PGC-1α as detected by immunofluorescence and Western blot. (E) Percent survival in cells exposed to H_2_O_2_ (350 µm, 1 h). Values represent means ± SEM; * indicates significant difference in survival compared to mock treatment and negative control (*P* < 0.05). (F) Mitochondrial membrane potential detected using Mitotracker Red in cells exposed to H_2_O_2_ (350 µm, 1 h). (G) Western blot to detect GFP or PGC-1α in equivalent relative amounts of protein from cytoplasmic and nuclear extracts, a four times equivalent of the insoluble nuclear pellet was loaded to facilitate detection. (H) Immunofluorescent detection of PGC-1α in cells over-expressing GFP-tagged PGC-1α and grown in the absence or presence of H_2_O_2_ (350 µm, 45 min) using anti-GFP or anti-PGC-1α antibodies.

We first examined PGC-1α function and regulation using a cell culture model. As shown, extra copies of PGC-1α conferred increased resistance to oxidative stress in mouse fibroblasts (Fig. 1A), in agreement with previous studies (Valle *et al*., 2005; St-Pierre *et al*., 2006). In addition, over-expression of PGC-1α causes an increase in mitochondrial membrane potential (Valle *et al*., 2005). If the increase in stress resistance of the PGC-1α over-expressing cells were because of the difference in mitochondrial membrane potential, we would expect regulation of mitochondrial membrane potential to be a component of the stress response. As shown, live cell staining with MitoTraker Red indicated that mitochondrial membrane potential was elevated within 1 h following exposure to low-intensity oxidative stress (Fig. 1B). The increase in mitochondrial membrane potential with hydrogen peroxide treatment was confirmed using JC1 staining. JC1 aggregates fluoresce at a higher wavelength than free JC1 molecules; because JC1 accumulates in proportion to the membrane potential, the ratio of the higher (Fl2) to lower (Fl1) wavelength is an indicator of membrane potential. In agreement with the increase in membrane potential, there was an increase in protein levels of COX IV, a subunit of the mitochondrial electron transport system and a target of PGC-1α transcriptional activity (Fig. 1C). Although the free radical scavengers MnSOD and catalase are known to be induced by PGC-1α 4 h following treatment with hydrogen peroxide (St-Pierre *et al*., 2006), levels of both proteins were unaltered in this 1 h time period. This result suggests that the effect of PGC-1α in promoting survival in the early response to oxidative stress is not because of the induction of the antioxidant defence. Knock down of PGC-1α levels by RNAi (Fig. 1D) increased cellular sensitivity to oxidative stress (Fig. 1E) and prevented the stress-mediated increase in mitochondrial membrane potential (Fig. 1F).

### Changes in intracellular PGC-1α distribution and acetylation status

To determine the cellular localization of PGC-1α, we used a strain over-expressing GFP-tagged PGC-1α. Antibodies against GFP or the N-terminal region of PGC-1α reveal that PGC-1α is localized both in the cytoplasm and in the nucleus (Fig. 1G). These data are confirmed by immunofluorescence (Fig. 1H). The observed difference in nuclear distribution as seen by subcellular fractionation is possibly because of over-expression of GFP-tagged PGC-1α. By immunofluorescence, both antibodies detect nuclear accumulation of PGC-1α following exposure to oxidative stress. These data suggest that PGC-1α subcellular distribution is shifted to the nucleus in the early response to oxidative stress where it regulates mitochondrial function.

**Fig. 2:**
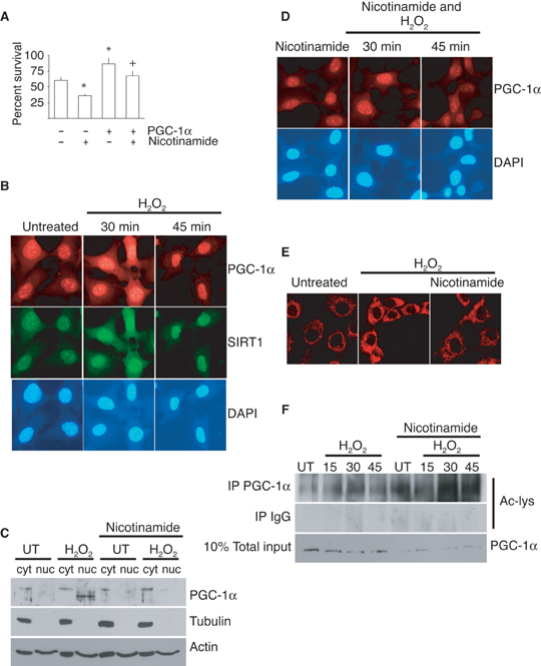
SIRT1 regulates PGC-1α subcellular localization and activity in response to stress. (A) Percent survival of cells after exposure to H_2_O_2_ (350 µm, 1 h) with or without nicotinamide (10 mm); values represent means ± standard error of the mean; * indicates significant difference in survival compared to control cells; + indicates significant difference in survival compared to peroxide-treated control cells (*P* < 0.05). (B) Immunofluorescent detection of PGC-1α and SIRT1 in cells following treatment with H_2_O_2_ (350 µm). Nuclei were visualized with DAPI stain. (C) PGC-1α in cytoplasmic (cyt) and nuclear (nuc) subcellular fractions in cultured cells after treatment with H_2_O_2_ (350 µm, 45 min), with or without nicotinamide (10 mm). (D) Immunofluorescent detection of PGC-1α in nicotinamide-treated cells (10 mm) after treatment with hydrogen peroxide (350 µm, 45 min). (E) Mitotracker Red detection of mitochondrial membrane potential after exposure to H_2_O_2_ (350 µm, 1 h) in nicotinamide- (10 mm) treated cells. (F) Detection of acetylated PGC-1α by Western in immunoprecipitates from cells following exposure to hydrogen peroxide (350 mm), with and without nicotinamide (10 mm).

SIRT1 NAD-dependent deacetylase is a member of the sirtuin family that has previously been shown to play a role in the oxidative stress response (Longo & Kennedy, 2006). SIRT1 functionally interacts with and deacetylates PGC-1α, promoting its transcriptional activity (Nemoto *et al*., 2005; Rodgers *et al*., 2005; Baur *et al*., 2006; Lagouge *et al*., 2006). To test if SIRT1 is also regulating PGC-1α activity in response to stress, cells were exposed to hydrogen peroxide in the absence or presence of nicotinamide, a potent sirtuin inhibitor (Bitterman *et al*., 2002; Avalos *et al*., 2005). Nicotinamide permits the immediate inhibition of SIRT1 activity and avoids the necessity to culture cells in the absence of SIRT1. As shown, stress resistance was impaired in wild-type cells in the presence of nicotinamide, but in cells over-expressing PGC-1α, the inhibitory effect was significantly reduced (Fig. 2A). These data place PGC-1α downstream of SIRT1 in the stress response because elevated levels of PGC-1α can compensate for the negative effect of nicotinamide on cell survival.

Prompted by these findings, we set out to explore the immediate response of PGC-1α to a low-intensity stress in cells that are not genetically manipulated. Under normal conditions, PGC-1α was detected both in the nucleus and the cytoplasm. Following oxidative stress, SIRT1 and PGC-1α accumulated in the nucleus and were colocalized (Fig. 2B). This distribution pattern was transient and, 1 h after exposure to stress, PGC-1α localization returned to that of untreated cells. Subcellular fractionation revealed that in the presence of nicotinamide, PGC-1α failed to accumulate in the nucleus (Fig. 2C), and this was confirmed by immunofluorescence (Fig. 2D). Under these conditions, the stress-induced elevation of mitochondrial membrane potential was impaired (Fig. 2E). These data indicate that SIRT1 regulates a PGC-1α-dependent increase in mitochondrial membrane potential in response to stress, and that regulation of mitochondrial function is critical to survival. To test if SIRT1 regulation of PGC-1α was a direct or an indirect effect, we probed for acetylation of PGC-1α in immunoprecipitates from cells exposed to hydrogen peroxide. As shown, PGC-1α acetylation was increased within 15 min of exposure to stress, with subsequent deacetylation by 45 min (Fig. 2F). In the presence of nicotinamide, PGC-1α was not deacetylated. The timing of modification coincides with PGC-1α accumulating in the nucleus, and when deactylation is prevented PGC-1α fails to localize appropriately. This suggests that the deacetylation of PGC-1α by SIRT1 is required to sequester it in the nucleus. These data demonstrate that PGC-1α activity is regulated by altering subcellular localization in response to stress in cells in culture.

### Nuclear degradation of PGC-1α

**Fig. 3:**
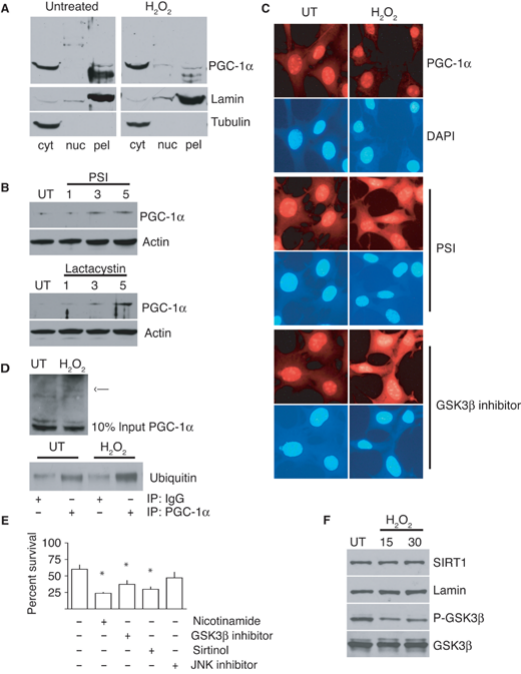
PGC-1α is targeted for GSK3β-dependent proteasomal degradation in response to oxidative stress. (A) Detection of PGC-1α protein by Western blot in subcellular fractions from cells 1 h after treatment with hydrogen peroxide (350 µm). (B) Detection of PGC-1α protein by Western blot in whole cell lysates from cells treated with proteasomal inhibitors PSI (10 µm) and lactacystin (10 µm) for the indicated times in hours. (C) Immunofluorescent detection of PGC-1α after treatment with hydrogen peroxide (350 µm, 45 min); cells were grown under normal conditions or with proteasomal (PSI 10 µm) or GSK3β (GSK3β inhibitor VIII 20 µm) inhibitors prior to and during stress. (D) Detection of ubiquitinated species by Western blot of PGC-1α immunoprecipitates from untreated and hydrogen-peroxide-treated cells (350 µm, 45 min). (E) Percent survival of cells after exposure to hydrogen peroxide (350 µm, 1 h); cells were grown under normal conditions or pre-incubated with nicotinamide (10 µm), GSK3β Inhibitor VII (20 µm), sirtinol (25 µm) or c-Jun N-terminal kinase (JNK) inhibitor II (25 µm); values represent means ± standard error of the mean; * indicates significant difference in survival compared to peroxide-treated control cells (*P* < 0.05). (F) Western blot detection of SIRT1, phospho-GSK3β and GSK3β in extracts taken at the times indicated in minutes from cells exposed to hydrogen peroxide (350 µm).

The transient nature of PGC-1α accumulation in the nucleus led us to ask how stress subsequently resets PGC-1α distribution to the cytoplasm. There are two most likely explanations: (i) PGC-1α is sequestered in the nucleus and, following transcriptional activation of target genes, is released back to the cytoplasm; and (ii) PGC-1α is sequestered in the nucleus but now, following transcriptional activation, the nuclear pool is degraded, and the cytoplasmic pool is replenished by transcriptional activation of the PGC-1α gene. To test this, we looked for evidence of nuclear degradation of PGC-1α. Subcellular fractionation revealed lower molecular weight forms and reduced levels of PGC-1α in the nuclear pool 1 h following stress (Fig. 3A). One of the principal mechanisms for specific depletion of proteins is targeted degradation by the proteasome. In agreement with a recent study, PGC-1α levels are increased in the presence of proteasomal inhibitors PSI and lactacystin, indicating that it is a target of the proteasome under normal culture conditions (Fig. 3B) (Sano *et al*., 2007).

In the presence of proteasomal inhibitor with subsequent exposure to hydrogen peroxide (45 min), immunofluorescent staining for PGC-1α was further elevated, and bright intranuclear foci containing PGC-1α were detected (Fig. 3C). The process of targeting proteins for degradation by the proteasome involves poly-ubiquitination of the target by specific ligases. Western blot detects high-molecular-weight bands in PGC-1α lysates following exposure of cells to hydrogen peroxide (45 min) (Fig. 3D). In addition, ubiquitin was detected in PGC-1α immunoprecipitates, and the ubiquitinated species was enriched in precipitates from cells exposed to hydrogen peroxide. These data indicate that PGC-1α is a substrate for degradation by the proteasome, and that turnover of PGC-1α protein is enhanced under oxidative stress conditions.

### Nuclear degradation of PGC-1α in response to stress requires GSK3β-dependent phosphorylation

Prior to ubiquitination and degradation, the target protein is usually ‘tagged’ by phosphorylation allowing it to be recognized by the ubiquitination apparatus. GSK3β has been previously associated with the stress response where it regulates levels of β-catenin, and as a result, activity of the forkhead transcription factor Foxo4 (Essers *et al*., 2005). Phosphorylation of β-catenin by GSK3β targets it for degradation by the proteasome (Aberle *et al*., 1997). We asked if GSK3β might be also involved in regulating PGC-1α stability. In the presence of GSK3β inhibitor and after 45 min of exposure to hydrogen peroxide, PGC-1α immunofluorescent staining was increased (Fig. 3C). Furthermore, cellular survival in response to oxidative stress was diminished in the presence of GSK3β inhibitor (Fig. 3E). GSK3β activity is regulated by inhibitory phosphorylation (Sutherland *et al*., 1993). Western analysis reveals that levels of GSK3β phosphorylation were reduced within 15 min of exposure to oxidative stress, indicating that it becomes activated (Fig. 3F).

**Fig. 4:**
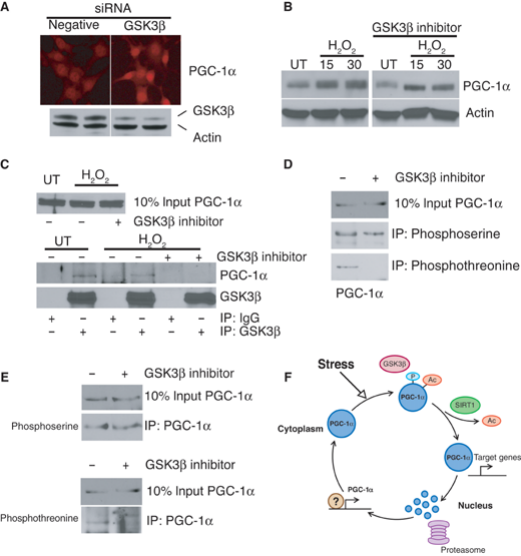
GSK3β-dependent phosphorylation of peroxisome proliferator-activated receptor-γ co-activator 1α (PGC-1α) in reponse to stress. (A) Immunofluorescent detection of PGC-1α in cells 24 h following siRNA with negative control or GSK3β-specific oligonucleotides. Knock down of GSK3β detected by Western blot (lower panel). (B) Western blot detection of PGC-1α in extracts taken at the indicated times in minutes following hydrogen peroxide treatment (350 µm); cells were grown under normal conditions or in the presence of GSK3β inhibitor VII (20 µm) prior to and during stress. (C) Detection of PGC-1α by Western blot of GSK3β immunoprecipitates from untreated or hydrogen-peroxide-treated cells (350 µm, 15 min); cells were grown in the absence or presence of GSK3β inhibitor VII (20 µm). (D) Detection of PGC-1α in phosphoserine and phosphothreonine immunoprecipitates from hydrogen-peroxide-treated cells (350 µm, 15 min); cells were grown in the absence or presence of GSK3β inhibitor VII (20 µm). (E) Detection of phosphoserine and phosphothreonine phosphorylated species by Western blot of PGC-1α immunoprecipitates from hydrogen-peroxide-treated cells (350 µm, 15 min); cells were grown in the absence or presence of GSK3β inhibitor VII (20 µm). (F) Model of PGC-1α activation in response to oxidative stress.

To confirm that GSK3β plays a role in PGC-1α processing, we generated cells with reduced levels of GSK3β using siRNA. In the absence of any other treatment, PGC-1α staining is elevated 24 h after knockdown of GSK3β, and the increase is most evident in the nuclei (Fig. 4A). To test whether GSK3β might be directly involved in PGC-1α processing, we examined PGC-1α protein levels by Western blot. Within 15 min of exposure to hydrogen peroxide, a shift in PGC-1α protein migration that is characteristic of phosphorylation was observed (Fig. 4B). In the presence of GSK3β inhibitor, this shift is no longer observed, indicating that PGC-1α may be a direct target of GSK3β.

To investigate this further, we conducted immunoprecipitaion experiments using extracts from cells grown in the absence or presence of GSK3β inhibitor and treated with hydrogen peroxide for 15 min. Immunoprecipitation of GSK3β coprecipitated PGC-1α, and this association was impaired in the presence of GSK3β inhibitor (Fig. 4C). These data indicate that PGC-1α and GSK3β physically associate during the stress response in a manner that is dependent on GSK3β activity. Immunoprecipitation using phospho-specific antibodies indicated that PGC-1α is phosphorylated at serine and threonine residues in this time frame (Fig. 4D). In the presence of GSK3β inhibitor, PGC-1α is co-immunoprecipitated using phosphoserine-specific antibodies, but not when using phosphothreonine-specific antibodies. In reciprocal experiments, a band was detected in PGC-1α immunoprecipitates using anti-phosphoserine- or anti-phosphothreonine-specific antibodies, but in the presence of GSK3β inhibitor anti-phosphothreonine did not detect PGC-1α (Fig. 4E). The consensus site for GSK3β phosphorylation is S/TxxxS/T (Fiol *et al*., 1987); phosphorylation of the C-terminal residue of the consensus by a priming kinase is a requisite for GSK3β-dependent phosphorylation at the N-terminal residue. These data indicate that serine phosphorylation of PGC-1α is at least in part independent of GSK3β, and threonine phosphorylation is GSK3β dependent.

Based on these data, we propose a model for PGC-1α regulation in the early response to stress (Fig. 4F). A key component of our model is its dependence on initial conditions prior to PGC-1α transcriptional activity. Upon exposure of the cells to hydrogen peroxide, GSK3β is activated and phosphorylates PGC-1α. As a result, PGC-1α is targeted for intranuclear proteasomal degradation. Now, when PGC-1α is deacetylated by SIRT1 and sequestered in the nucleus, a sustained effect on expression of target genes involved in the mitochondrial electron transport system is prevented. Overall levels of PGC-1α are not altered within this time frame as degradation of the nuclear pool is offset by increased transcription of PGC-1α gene.

### *In vivo* studies

**Fig. 5:**
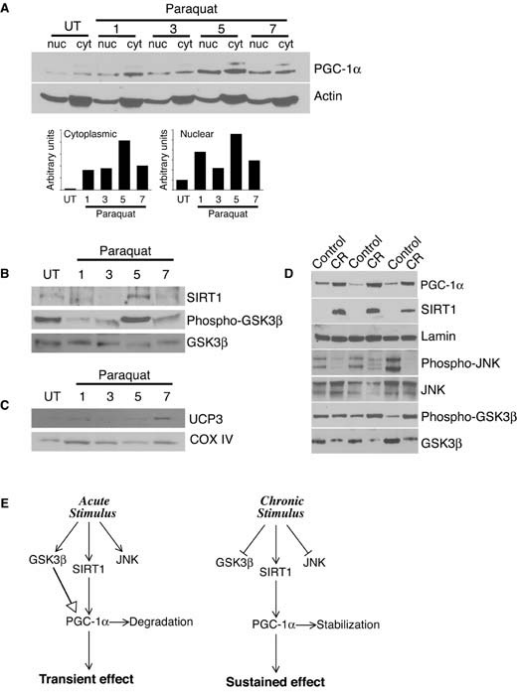
PGC-1α is regulated by SIRT1 and GSK3β during oxidative stress and calorie restriction (CR) in mice *in vivo*. (A) Western detection of PGC-1α in nuclear (nuc) and cytoplasmic (cyt) subcellular fractions from skeletal muscle of 5-month-old mice taken at indicated hours following exposure to paraquat (50 mg kg^−1^ body weight). Densitometric analysis of normalized PGC-1α levels detected in cytoplasmic and nuclear fractions at indicated times. (B) Western detection of SIRT1, GSK3β and phospho-GSK3β in whole tissue homogenates of skeletal muscle of 5-month-old mice taken at indicated hours following exposure to paraquat (50 mg kg^−1^ body weight). (C) Western detection of UCP3 and COX IV in cytoplasmic fraction of skeletal muscle of 5-month-old mice taken at indicated hours following exposure to paraquat (50 mg kg^−1^ body weight). (D) Western detection of SIRT1, phospho-c-Jun N-terminal kinase (JNK), JNK, phospho-GSK3β and GSK3β in white adipose tissue from control (Control) and restricted (CR) 10-month-old mice. (E) Model describing regulation of PGC-1α that permits transient or sustained effects on PGC-1α activity.

We next looked for evidence of PGC-1α regulation by this mechanism *in vivo* in mice. Mice were exposed to the oxidative stressor paraquat, and tissue was harvested and processed for subcellular fractionation at the indicated time points (Fig. 5A). PGC-1α accumulated in the nuclear fraction in mouse skeletal muscle following exposure to paraquat. Densitometric analysis revealed that cellular redistribution of PGC-1α was biphasic, with an initial increase in nuclear accumulation at 1 h and a second further increase at 5 h post-treatment. Western blot analysis demonstrated that GSK3β levels were not changed following stress (Fig. 5B). However, after 1 h of treatment, phospho-GSK3β levels were reduced, indicating activation of GSK3β. After 5 h, levels of phospho-GSK3β were increased beyond basal levels observed in the untreated animals. SIRT1 levels were increased at 5 h following treatment. Consistent with our proposed model, the biphasic response of PGC-1α regulators was reflected in the pattern of accumulation of PGC-1α in the nucleus, and in the levels of COX IV and UCP3 which are targets of PGC-1α (Fig. 5C).

We next wished to determine if this mechanism of PGC-1α regulation was peculiar to the stress response or if these factors identified in the stress response could regulate PGC-1α in a more general manner. As shown, GSK3β and SIRT1 were both regulated by CR in white adipose tissue (Fig. 5D). Protein levels of SIRT1 were increased in adipose tissue from restricted animals compared to controls. At the same time, GSK3β levels were reduced and the relative level of phosphorylation was increased, indicating that GSK3β is less active. SIRT1 positively regulates PGC-1α localization and transcriptional activity, and inhibition of GSK3β would stabilize PGC-1α against proteasomal degradation, consistent with the observed increase in PGC-1α levels and increased expression of multiple PGC-1α gene targets in adipose tissue (Higami *et al*., 2004). We next wished to determine if other factors associated with the stress response in cells in culture also play a role in CR. The stress-activated kinase c-Jun N-terminal kinase (JNK) has been linked to insulin resistance, and abnormally elevated JNK activity is associated with obesity (Hirosumi *et al*., 2002). The fact that CR confers increased insulin sensitivity and causes a 70% reduction in fat mass suggested JNK as a potential candidate regulated by CR. As shown, CR negatively regulated JNK activity (but not amount) in adipose tissue, as decreased levels of phosphorylation are indicative of reduced kinase activity. These data demonstrate that CR impacts stress response pathways and suggests that the link between longevity and stress resistance is through a shared mechanism.

The subcellular distribution of PGC-1α provides a mechanism for rapidly altering its transcriptional activity, and PGC-1α intranuclear stability determines the duration of transcriptional activity (Fig. 5E). In the case of the acute stress response, changes in mitochondrial function occur rapidly. The mechanistic basis of this observation is that stress activates GSK3β and SIRT1 resulting in PGC-1α activation and subsequent degradation. In the case of CR (a chronic stress), induction of SIRT1 and inhibition of GSK3β activate and stabilize PGC-1α, resulting in a sustained increase in expression of genes involved in the mitochondrial energy metabolism.

## Discussion

Mitochondrial function declines with age in humans (Short *et al*., 2005), and a decline in the expression of components of the electron transport chain is a hallmark of aging across species (Zahn *et al*., 2006). However, the extent of the contribution of mitochondrial function to the onset of age-related pathologies like diabetes and heart disease is not yet clear. Mitochondrial dysfunction has pleiotropic effects in mammals, and there is evidence to suggest that changes in mitochondrial function can implement large-scale metabolic changes *in vivo* (Chan, 2006).

In this study, we have shown that increased copies of PGC-1α are sufficient to exert an effect on mitochondrial function through increase in mitochondrial membrane potential, and that PGC-1α is required for the stress-induced increase in mitochondrial membrane potential. These findings indicate that PGC-1α plays a role in mitochondrial adaptation and that regulation of mitochondria is a component of the stress response. Why the cellular response to oxidative stress alters mitochondrial function is a matter of speculation: the changes in membrane potential could reflect (i) a response to increased energy demand as cellular survival pathways are induced; (ii) a change in flux of endogenous mitochondrial-derived reactive oxygen species (ROS) implemented as a protective mechanism to minimize mitochondrial damage; or (iii) the alteration in mitochondrial function is itself a secondary signal in the response to stress. While studies in isolated mitochondria indicate that increased ROS causes a decrease in membrane potential, the experiments described here differ significantly in that the cells are intact upon exposure to hydrogen peroxide retaining the possibility for extra-mitochondrial signalling and nuclear response.

We describe a mechanism of PGC-1α regulation through post-translation modification by SIRT1 and GSK3β, that permits transient or sustained effects on mitochondrial function. In addition, we have provided evidence in support of our proposed model in animal studies where we demonstrate that the same regulators are involved in the *in vivo* stress response and in CR. In exploring the relationship between SIRT1 and PGC-1α, we utilized the inhibitors nicotinamide and sirtinol. These inhibitors are not specific to SIRT1, but also influence other members of the sirtuin family (Denu, 2005). Given the established relationship between SIRT1 and PGC-1α and the colocalization of these proteins in response to oxidative stress, it is likely that the effect of these inhibitors on PGC-1α acetylation is because of inhibition of SIRT1. However, we cannot rule out the possibility that other sirtuins (Michishita *et al*., 2005) play a role in the stress response and may contribute to the effect of these inhibitors on cellular survival.

We have shown that PGC-1α stability is regulated by GSK3β, which targets PGC-1α for intranuclear proteasomal degradation. We find that PGC-1α accumulates in bright foci in the nucleus in the presence of proteasomal inhibitors. The same staining pattern is also observed in the presence of GSK3β inhibitor. An independent study has shown that these bright foci costain with PML bodies and are associated with the insoluble nuclear fraction (Sano *et al*., 2007). Interestingly, in our over-expression study using GFP-tagged PGC-1α, the nuclear distribution of the over-expressed tagged PGC-1α differs from that of the endogenous protein. Nuclear GFP–PGC-1α is detected in the soluble and insoluble fractions, whereas the endogenous protein is predominately found in the insoluble fraction. Immunofluorescence using either anti-GFP of anti-PGC-1α antibodies detected a similar staining pattern in untreated cells, but a subtle difference in the oxidative-stress-treated cells (Fig. 1). The areas of nuclear exclusion are evident in cells probed with anti-GFP following stress, but not in the cells probed with anti-PGC-1α, raising the possibility that the tagged protein is not being processed in the same manner as the endogenous protein.

Regulation of transcription factor activity through subcellular localization and protein stability provides mechanism whereby the expression of key genes is facilitated for a specific duration (Kodadek *et al*., 2006). It is possible that transcription factor activation that is combined with degradation could influence the behaviour of the transcriptional activator, as in the case of the SAGA complex where activity is stimulated by proteasomal association (Lee *et al*., 2005). We describe an initial transient activation of PGC-1α in response to stress, while a sustained increase in PGC-1α levels is detected at a later time point (St-Pierre *et al*., 2006). The transcriptional targets appear to be specialized for either the early or later response, as COX IV levels are increased in the early response, but unlike the later time frame, the expressions of antioxidant scavengers MnSOD and catalase are not increased. The *in vivo* experiment described here (Fig. 5) revealed a biphasic nature to the regulation of PGC-1α localization in skeletal muscle from mice treated with paraquat, and this was reflected in the difference in timing of induction of COX IV and UCP3 proteins. It will be interesting to determine how PGC-1α gene target specificity is regulated under these conditions, and if its association with the proteasome is a determining factor.

There is evidence to suggest that CR induces specific pathways that promote longevity. For example, in yeast, CR and numerous low-intensity stressors associated with longevity activate a common pathway to influence lifespan (Anderson *et al*., 2003). Here, we show that PGC-1α transcriptional activity is induced in the oxidative stress response and CR through a shared mechanism, suggesting that in mammals, regulation of mitochondrial function is a key element in both cellular survival and longevity. We propose that mitochondrial plasticity may be critical for maintaining cell viability and in orchestrating the program of aging retardation by CR, raising the possibility that loss of mitochondrial plasticity is an underlying cause of aging.

## Experimental procedures

### Animal treatments

Wild-type male C57B16 mice were housed under controlled, specific pathogen-free conditions at the Shared Aging Rodent Facility at Madison VA Geriatric, Research, Education, and Clinical Center. The mice were cared for in accordance with the Institutional Animal Care and Use Committee at UW Madison. To control calorie intake, the mice were housed singly and fed less than *ad libitum* intakes every other day. All mice were euthanized by cervical dislocation. The control animals were fed 98 kcal week^−1^ of AIN-M semipurified diet (Bioserve, Frenchtown, NJ, USA), which is about 90% of the average *ad libitum* intake for these mice. The restricted mice were fed 58 kcal week^−1^ (a 41% reduction) from 8 weeks of age. The restricted diet was nearly isocaloric to the control diet, but was enriched in proteins, vitamins and minerals to avoid malnutrition. Under this regimen of controlled intake, animals ingest all the allocated food, and the calorie intake is precisely known. For the oxidative stress experiment, the mice were treated with paraquat (single intraperitoneal injection 50 mg kg^−1^) and sacrificed at 1, 3, 5 and 7 h after exposure. Tissues were collected, snap frozen in liquid nitrogen and stored at –80 °C until further processing by microarray or immunoblotting.

### Cell culture

NIH3T3 cells (ATCC, Rockville, MD, USA) were cultured in Dulbecco's modified Eagle's medium with 10% foetal bovine serum and antibiotics. Transfections were performed with Lipofectamine (Invitrogen, Carlsbad, CA, USA) according to the manufacturer's instructions using pcDNA3.1 and pcDNA–PGC-1α (gift from Dr D Kelly, Washington University, St Louis, MI, USA). Clones were isolated, and expression of PGC-1α was confirmed by Western blot. The GFP-tagged PGC-1α was generated by polymerase chain reaction cloning the PGC-1α cDNA from pcDNA–PGC1α into pcDNA3.1/NT–GFP–TOPO fusion vector (Invitrogen). Knock down of PGC-1α or GSK3β was performed by RNA interference using Silencer pre-designed siRNAs (Ambion, Austin, TX, USA), and introduced into cells by electroporation using siPORT electroporation kit (Ambion). To determine sensitivity to oxidative stress, subconfluent cells were exposed to hydrogen peroxide (350 µm) in serum-free media for up to 1 h. For inhibitor/viability experiments, cells were grown 1 h pre-incubation in the presence of nicotinamide [(Sigma, St. Louis, MO, USA), 10 mm], PSI proteasomal inhibitor [(Calbiochem, EMD/Merck, Darmstadt, Germany), 10 µm], or 2 h pre-incubation GSK3β inhibitor VII (Calbiochem, 20 µm), Sirtinol (Calbiochem, 25 µm) and JNK inhibitor II (Calbiochem, 25 µm) prior to and during stress. Cell viability was determined by measuring fluorescence from cells in phosphate-buffered saline (PBS) with carboxyfluorescein diacetate (Sigma, 400 nm) on a LS50B Perkin Elmer luminescence spectrometer (Perkin Elmer, Waltham, MA, USA) (λ_ex_ 480 nm, λ_em_ 525 nm). Experiments were performed in triplicate, and values from four wells each were corrected for background fluorescence and normalized against identically treated cells without peroxide exposure. For localization and live imaging studies, cells were grown on cell-culture-treated cover slips (Fisher Scientific, Thermo Fisher Scientific, Waltham, MA, USA). For protein stability experiments, cells were incubated in either PSI (10 µm) or lactacystin (Calbiochem, 10 µm) for the indicated times. For mitochondrial membrane potential measurement by JC-1 staining, ∼1 × 10^6^ cells in triplicate were treated in the absence or presence of hydrogen peroxide 1 h and stained with presonicated JC-1 (Sigma, 1 µg mL^−1^) for 15 min. Cells were trypsinized, resuspended in PBS and fluorescence detected at λ_ex_527 nm and λ_ex_590 nm using Perkin Elmer LS50B.

### Protein preparation and immunodetection

Proteins from mouse tissues and cultured cells were extracted in modified RIPA buffer (Tris–HCL 50 mm, pH 7.4; NP-40 1%; Na-deoxycholate 0.25%; NaCl 150 mm; ethylenediaminetetraacetic acid 1 mm) containing protease inhibitors (Sigma) and where indicated phosphatase inhibitors (Sigma). Proteins were detected by immunoblotting using standard techniques. Antibodies used were PGC-1α, SIRT1, lamin A, ubiquitin (Santa Cruz Biotechnology, Santa Cruz, CA, USA), GSK3β (Biodesign, Meridian Life Science. Inc., Cincinnati, OH, USA), COX IV (Abcam Inc., Cambridge, MA, USA), GFP, MnSOD, catalase (Genetex Inc., San Antonio, TX, USA), GSK3β, phospho-GSK3β, JNK, phospho-JNK, Ac-lys (Cell Signaling Technology), actin and tubulin (Sigma). Subcellular fractionation was performed using nuclear/cytoplasmic fractionation kit (Biovision, Mountain View, CA, USA). For immunoprecipitation, 500 µg of extract was incubated overnight with control IgG or specific primary antibody; antibodies were precipitated with Protein A or Protein G Agarose beads (Santa Cruz Biotechnology). Immunoprecipitates were analysed by Western blot. Densitometric analysis was performed using NIH ImageJ software (http://rsb.info.nih.gov/ij/); cytoplasmic and nuclear band densities normalized against actin loading control and were analysed separately.

### Localization and live cell imaging

Cellular localization was analysed by immunofluorescence using standard techniques. Following exposure to stress, cells were fixed (3.7% formaldehyde, 15 min) at the indicated times. Cells were incubated overnight in primary antibody, and cellular distribution of proteins was visualized using fluorescent-tagged secondary antibodies (Vector Laboratories, Burlingame, CA, USA). For live imaging, cells were exposed to stress in media lacking phenol red, incubated in Mitotracker Red [(Molecular Probes, Invitrogen, Carlsbad, CA, USA), 300 mm] for 10 min and washed with PBS prior to analysis. All images were captured using uniform exposure settings on a Leica DMLB microscope fitted for epi-fluorescence (Leica Microsystems, Wetzlar, Germany), with a Spot Insight Color camera (Diagnostic Instruments, Sterling Heights, MI, USA) and Spot 3.3.1 software.

### Statistical analysis

Effects of treatments were analysed by two-tailed *t*-test assuming equal variance. Differences were considered statistically significant at *P* < 0.05.
